# Inhibitors of the Hepatitis C Virus Polymerase; Mode of Action and Resistance

**DOI:** 10.3390/v7102868

**Published:** 2015-09-29

**Authors:** Auda A. Eltahla, Fabio Luciani, Peter A. White, Andrew R. Lloyd, Rowena A. Bull

**Affiliations:** 1 Systems Medicine, Inflammation and Infection Research Centre, School of Medical Sciences, Faculty of Medicine, University of New South Wales, Sydney 2052, Australia; a.eltahla@unsw.edu.au (A.A.E.); luciani@unsw.edu.au (F.L.); a.lloyd@unsw.edu.au (A.R.L.); 2 School of Biotechnology and Biomolecular Sciences, Faculty of Science, University of New South Wales, Sydney 2052, Australia; p.white@unsw.edu.au

**Keywords:** hepatitis C virus, polymerase inhibitor, direct-acting antiviral, NS5B, antiviral resistance, RNA dependent RNA polymerase

## Abstract

The hepatitis C virus (HCV) is a pandemic human pathogen posing a substantial health and economic burden in both developing and developed countries. Controlling the spread of HCV through behavioural prevention strategies has met with limited success and vaccine development remains slow. The development of antiviral therapeutic agents has also been challenging, primarily due to the lack of efficient cell culture and animal models for all HCV genotypes, as well as the large genetic diversity between HCV strains. On the other hand, the use of interferon-α-based treatments in combination with the guanosine analogue, ribavirin, achieved limited success, and widespread use of these therapies has been hampered by prevalent side effects. For more than a decade, the HCV RNA-dependent RNA polymerase (RdRp) has been targeted for antiviral development. Direct acting antivirals (DAA) have been identified which bind to one of at least six RdRp inhibitor-binding sites, and are now becoming a mainstay of highly effective and well tolerated antiviral treatment for HCV infection. Here we review the different classes of RdRp inhibitors and their mode of action against HCV. Furthermore, the mechanism of antiviral resistance to each class is described, including naturally occurring resistance-associated variants (RAVs) in different viral strains and genotypes. Finally, we review the impact of these RAVs on treatment outcomes with the newly developed regimens.

## 1. Introduction

Hepatitis C virus (HCV) is a significant human pathogen affecting nearly 3% of the world’s population, and is a leading cause of progressive chronic liver disease, potentially culminating in cirrhosis and hepatocellular carcinoma [1]. HCV is a small enveloped virus, 50–80 nm in diameter, with a positive sense, single stranded RNA genome (ssRNA) of ~9600 nucleotides. The RNA molecule contains a single open reading frame (ORF) but lacks a 5ʹ cap. Instead, translation is initiated in the cytoplasm through an internal ribosome entry site [2,3]. A single amino acid precursor polyprotein is translated at the endoplasmic reticulum (ER) and is then cleaved by host and viral proteases into 10 structural and non-structural (NS) proteins. The structural proteins of HCV are encoded by the 5ʹ terminus of the genome and include the core protein as well as the two glycoproteins, E1 and E2. These are followed by the NS proteins which include p7, NS2, NS3, NS4A, NS4B, NS5A and NS5B [4].

The polymerase of HCV, NS5B, exhibits a high mutation rate of approximately 10^−4^ substitutions per site [5,6]. This is combined with a very high rate of virion production in infected individuals (10^12^ virions per day [7]). As a result, within host, the HCV genome exists as a heterogeneous RNA population known as a quasispecies [8]. This heterogeneity contributes to a significant evolutionary advantage and provides the virus with the means to adapt to the host immune response and persist as a chronic infection [9]. At the human population level, HCV has undergone significant evolution resulting in the emergence of seven different genotypes (GT1–GT7) differing by approximately 35% at the nucleotide level [10]. These genotypes are further classified into “subtypes” (a, b, c, *etc.*), with about 20% inter-subtype divergence across the genome [11,12].

## 2. The RNA-Dependent RNA Polymerase (RdRp)

Soon after the discovery of HCV, the analysis of its amino acid sequence predicted the existence of a “replicase” protein based on the identification of a Gly-Asp-Asp (GDD) motif, a signature conserved sequence for RdRps of other RNA viruses [13]. *In vitro* analysis of the non-structural region of HCV genome also confirmed the biochemical activity of an RdRp that was proposed to mediate genome replication [14].The RdRp was later characterised as a 66 kDa protein and, like most of the HCV non-structural proteins, associates with cellular membranes. For the RdRp, this was mediated by a hydrophobic C-terminal amino acid tail [15]. However, the deletion of this hydrophobic “anchor” had little effect on the enzymatic activity *in vitro*, and recombinant, soluble RdRp could be produced efficiently in both insect cells and *Escherichia coli* [16,17,18]. This in turn has enabled extensive structural and functional studies of the RdRp, which has rapidly become the best characterised of the HCV enzymes.

**Figure 1:**
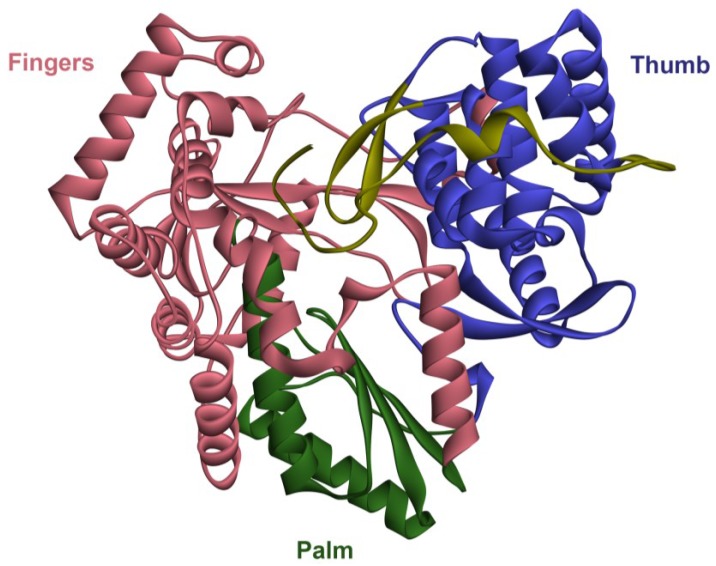
Crystal structure of the hepatitis C virus (HCV) RNA-dependent RNA polymerase (RdRp). The secondary structure of the HCV GT1b polymerase (Protein Data Bank (PDB) accession number 3FQL) is shown. The enzyme has a typical RdRp “right hand” structure with fingers (red) and thumb (blue) subdomains encircling the active site within the palm subdomain (green). The enzyme is typically crystallised in the shown “closed” conformation, thought to be associated with *de novo* RdRp activity, with the β-hairpin from the thumb domain and the C-terminal “linker” shown in yellow extending into the palm domain of the RdRp.

The crystallisation of the HCV RdRp revealed a canonical right-hand like structure, where the active site (GDD motif, also known as motif C) in the palm subdomain is fully encircled by an extensive interaction between the fingers and thumb subdomains [19,20,21] (Figure 1). The HCV RdRp shares some structural homology with other viral RdRps and reverse transcriptases (RTs), including the RdRp from the RNA bacteriophage phi6 [22]. The protein also harbours a conserved aspartic acid motif (motif A) that coordinates the binding of metal ions like Mg^2+^. The thumb subdomain of the HCV RdRp contains a β-hairpin loop insertion, which protrudes into the active site cavity (Figure 1). This loop is thought to influence the orientation of the newly synthesised RNA, and its position discriminates between different modes of RdRp activity [23]. Interestingly, an allosteric guanosine-5ʹ-triphosphate (GTP)-binding pocket has also been identified in the interface between the fingers and thumb subdomains [24]. The binding of GTP to this site has been implicated in facilitating conformational changes required for a processive RdRp [25].

The RdRp was originally crystallised in a “closed” conformation, which can only accommodate a single strand of RNA in the active site [19,20,21]. However, this did not explain how the enzyme could accommodate the double-stranded RNA being synthesised. *In vitro* experiments using recombinant RdRp show that the enzyme is capable of two biochemical activities. Firstly, the enzyme can catalyse RNA synthesis in a primer-dependent manner, extending from the 3ʹ-end of an RNA molecule [14,26]. However, the RdRp of HCV is also able to catalyse transcription through a primer-independent, or *de novo* mechanism, where the polymerase catalyses the formation of a dinucleotide molecule at the 3ʹ-end of the template, which could then be used as a primer [27,28,29]. This mechanism is non-deleterious for the viral genome, that is, the whole genome is copied from start to finish, and this mode of activity for the RdRp is believed to be how genome replication is initiated *in vivo* [28]. The closed conformation of the RdRp is thought to be associated with this *de novo* formation of the dinucleotide complementary to the 3ʹ-end of template RNA [30], whereas an open conformation is thought to represent the primer-extension activity of the HCV RdRp, and is associated with the displacement of the β-hairpin loop as well as a C-terminal segment, upstream of the transmembrane domain, called the linker (Figure 1) [23,31].

## 3. Therapies for HCV in the Past

The aim of treatment for patients chronically infected with HCV is to achieve a sustained virological response (SVR), defined as the absence of detectable HCV RNA from the blood 12 or 24 weeks after treatment is discontinued [32]. For more than a decade, the standard of care (SOC) involved a combination of pegylated interferon-α (Peg-IFN-α) and ribavirin (RBV) for 24 to 48 weeks, depending on the infecting HCV GT [32,33]. The HCV RNA level of the patient, known as the viral load, is monitored throughout the duration of the treament in order to determine the pattern of response to therapy. Response to such IFN-based therapy varies greatly amongst patients, and is predominantly affected by the HCV genotype and the presence or absence of cirrhosis. Overall, patients infected with GT2 and GT3 HCV are better respondents to the IFN-based therapy, with SVR rates up to 80% [34]. In contrast, the more prevalent GT1 is associated with a poorer response rate with less than 50% of patients achieving SVR [35]. Peg-IFN/RBV treatment is expensive, and almost all patients experience adverse effects [32]. The SOC for GT1 was modified with the licensing of the first generation direct-acting antivirals (DAA) in 2011—protease inhibitors (PI), telaprevir and boceprevir, administered in combination with Peg-IFN/RBV as a triple therapy. In 2014, the landscape for HCV therapy was significantly shifted with the approval of the first pan-genotypic antiviral for HCV; sofosbuvir, targetting the HCV RdRp and opening the door to IFN-free combination DAA therapies [36,37,38,39,40].

## 4. HCV RdRp as a Target for DAAs

**Figure 2:**
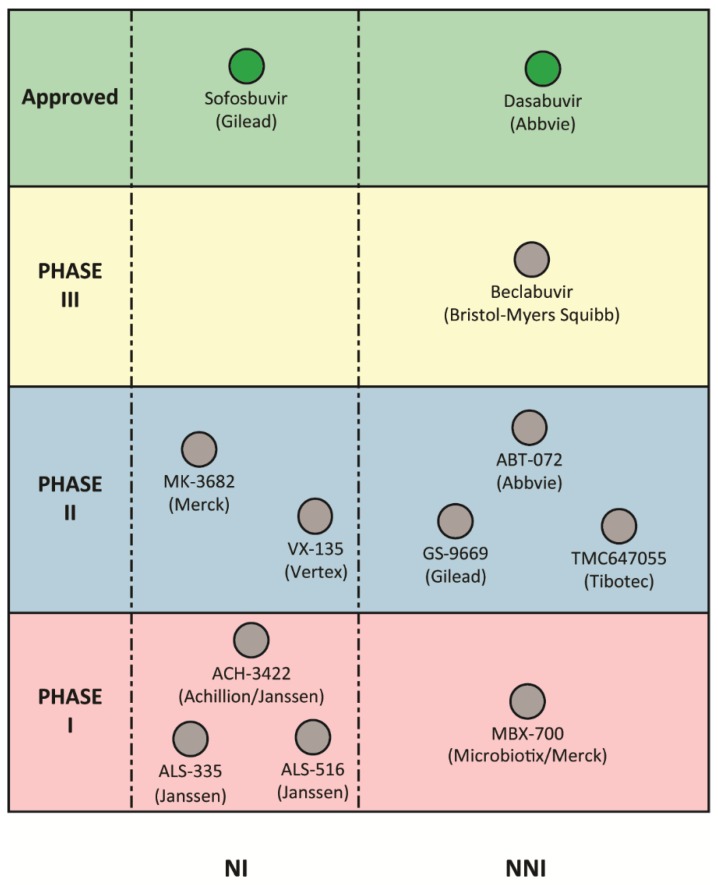
Direct-acting antivirals targeting the HCV RdRp in clinical development.A summary of DAAs targeting the HCV RNA dependent RNA polymerase (RdRp) in different stages of clinical trials is shown along with the developing companies. RdRp inhibitors are divided into nucleoside (NI) and non-nucleoside inhibitors (NNI). Compounds that are in different phases of clinical trials are shown as grey spheres, while those that have been approved for clinical use are shown as green spheres.

The RdRp has been a prime target for antiviral development, given its vital role in viral replication. Molecules that bind to and inhibit the HCV RdRp fall into two main categories: nucleoside and non-nucleoside inhibitors (Figure 2).

**Nucleos(t)ide inhibitors** (NI) are nucleoside/nucleotide analogues, which are incorporated into the nascent genome by the RdRp, and prevent further incorporation of incoming nucleotides [41]. HCV NIs possess a 3ʹ-hydroxyl group, and such molecules are classified as non-obligate chain terminators. It has therefore been proposed that these molecules cause chain termination by steric hindrance exerted through 2ʹ-C-methyl or 2ʹ-fluoro groups [42]. Both nucleoside and nucleotide analogues need to be phosphorylated into a triphosphate form in the cell to be active, however, nucleotide analogues bypass the first phosphorylation step. Since this first phosphorylation step is the rate-limiting stage for activation, nucleotide analogues result in higher concentrations of nucleoside triphosphates in the cell [43]. To reduce systemic exposure of these drugs, HCV NIs are developed as “prodrugs” which can be preferentially cleaved by hepatic enzymes.

As all NIs target the highly conserved active site of the polymerase, these inhibitors tend to be cross-genotypic [44]. Furthermore, although single amino acid mutations are sufficient to confer resistance to this class of inhibitors, these mutations appear to confer a fitness cost to viral replication [45,46]. The NI valopicitabine (NM283) was the first to demonstrate a proof-of-concept for the use of an RdRp inhibitor in the treatment of HCV [47], although its development was halted due to gastrointestinal side-effects [48,49].

**Figure 3:**
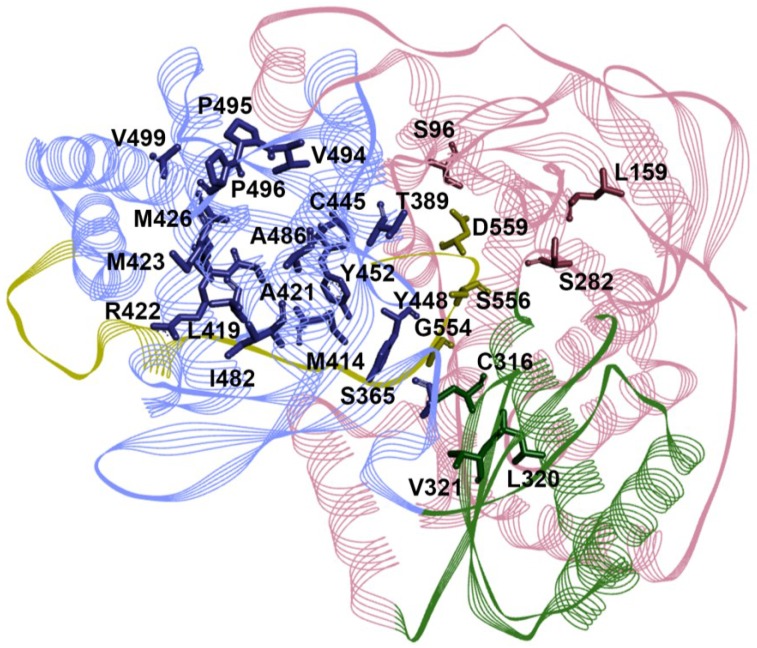
Positions of RdRp residues associated with resistance to HCV nucleoside (NI) and non-nucleoside inhibitors (NNI).The secondary structure of the HCV GT1b polymerase is coloured as in Figure 1, shown with the palm domain facing away. Known residues where substitutions confer resistance to NIs and NNIs are highlighted (Table 1).

The most advanced compound from this class of DAAs is sofosbuvir, which is a prodrug of 2'-F-2'-C-methyluridine monophosphate [50]. Sofosbuvir was initially licensed in 2013 for the treatment of GT1 and GT4 in combination with IFN/RBV, and in combination with RBV for GT2 and GT3. More recently, two IFN-free combination regimens containing sofosbuvir and an NS5A inhibitor or a protease inhibitor with RBV have been approved for GT1 patients after promising phase 3 trials [51,52]. In pre-clinical assessment of sofosbuvir, a Serine to Threonine substitution at position 282 of the RdRp (S282T, Figure 3) conferred a 10-fold resistance against sofosbuvir [53], a phenotype that has been observed for a number of HCV NIs [54]. *In vitro* analysis of the S282T mutant RdRps revealed that these enzymes have a decreased affinity for nucleoside analogues [54], and this substitution also resulted in a significant loss of replication fitness of HCV as assessed using replicon models [53]. Recent crystal structures of the HCV RdRp have revealed the conformational movement the S282 residue undergoes in order to interact with incoming nucleotide substrates and form a hydrogen bond [55]. Co-crystal structures with the active form of sofosbuvir also revealed the interruption of such molecular changes which keep the enzyme in its inactive apo form and prevent hydrogen bonding [55]. This provides further mechanistic evidence for the ability of S282T to confer resistance to NIs, and cause the loss of replicative fitness. In a recent meta-analysis of several sofosbuvir trials, substitutions at position 316 of the RdRp were found to be associated with failure of response to sofosbuvir, particularly for patients infected with GT1b [56]. Such substitutions were proposed to occupy larger spatial area within the catalytic site and therefore interfere with sofosbuvir entry. The same study also identified L159F and V321A as treatment-associated variants which are also associated with the lack of response to sofosbuvir treatment [56]. Interestingly, however, these variants had a minor effect on the potency of sofosbuvir when examined individually using cell culture models [57].

The NI mericitabine is a prodrug of 2ʹ-F-2ʹ-C-methylcytidine. Addition of mericitabine to Peg-IFN/RBV had only a modest effect on the SVR rates of infected patients when compared to IFN/RBV alone [58,59]. Interestingly while no S282T substitution, which is known to be associated with resistance to mericitabine, was detected in these studies, [45], a follow-up study of partial responders from these trials identified a pair of substitutions, L159F/L320F, near the active site of the RdRp (Figure 3) that, in combination, conferred up to 5.5-fold resistance to mericitabine and sofosbuvir when examined using replicon models [60]. In a recent trial of an IFN-free regimen containing mericitabine for patients with GT1 infection, only 25.0% and 64% of patients with GT1a and GT1b, respectively, reached SVR after 24 weeks [61]. While the majority of viral breakthrough cases in this trial were attributed to NS3 resistance mutations, mericitabine resistance mutations encoding S282T, L159F/L and L320F were detected at a very low frequency (3% of patients) after viral breakthrough, but none with viral relapse [61]. Mericitabine was recently halted from further clinical development.

Other NIs in phase 2 clinical development include VX-135, MK-3682 and ACH-3422 (Figure 2). Recent trials of VX-135 in combination with daclatasvir, an NS5A inhibitor, reported an SVR rate of 90% in patients with GT1 infection, with relapse once again associated with S282T substitutions [62]. A large number of other NIs have been developed, but halted mainly due to toxicity [46,63]. Interestingly, analysis of the off target effects of HCV NIs revealed that a subset of these molecules inhibit the activity of mitochondrial RNA polymerase, providing an explanation for their adverse effects in the clinical setting [64]. Nonetheless, HCV NIs will likely be a major component of future IFN-free, pan-genotype therapies based on the success of sofosbuvir.

Unlike NIs, **non-nucleoside inhibitors** (NNI) are inhibitors of the RdRp that are non-competitive with regards to the nucleotide substrate. Instead, the binding of these compounds to the RdRp inhibits conformational changes required for polymerase activity [65,66]. HCV NNIs target one of five allosteric sites on the RdRp; two of which are within the thumb subdomain (sites T1 and T2), and two within the palm subdomain (sites P1 and P2). More recently, a fifth site has been identified within the palm domain involving a unique interaction with the β-hairpin extending from the thumb domain (termed site P-β) [67,68] (Figure 1). The first generation of HCV NNIs have reported a relatively low barrier to resistance, with single mutations in the RdRp coding region associated with resistance to all identified HCV NNIs *in vitro* [69,70,71]. Furthermore, mutations conferring resistance to NNIs were detected in patients infected with all HCV GTs, even without NNI treatment [69]. The first proof-of-concept for the clinical use of HCV NNIs was with the P2 inhibitor nesbuvir [69]. The development of nesbuvir, however, was halted due to the induction of liver enzyme elevation.

**T1 inhibitors.** Thumb I (T1) is a subdomain to which compounds like benzimidazole and indole derivatives bind (e.g. deleobuvir, BMS-791325 and TMC647055). Deleobuvir (BI 207127) which progressed to phase 3 for the treatment of GT1 patients [72]. Amino acid substitutions P495, P496, and V499 (Figure 3, Table 1) were all associated with resistance to deleobuvir *in vitro* [73]. In a phase 3 trial of deleobuvir, administered as part of an IFN-free regimen, amino acid substitutions P495L/S/T were most commonly associated with viral breakthrough and relapse [74], and the development of deleobuvir was halted. TMC647055 is another indole derivative currently in phase 2 clinical trials that has been shown to have cross-genotypic activity when examined using *in vitro* replicon models, with the exception of GT2 [75]. Substitutions at NS5B amino acid residues L392 and P495 were associated with reduced sensitivity to TMC647055, with P495L showing a 371-fold reduction in potency [75]. Another indole derivative, beclabuvir, is currently in phase 3 clinical trials. *In vitro* characterisation of beclabuvir revealed potent activity against HCV in recombinant enzyme assays and cell culture models, however, reduced potency was also observed against GT2, and variable activity against GT6 viruses [76]. Similar to other molecules binding to T1, P495 substitutions (Figure 3) were associated with increased resistance to beclabuvir [76,77]. However another T1 RAV A421V, was identified upon treatment of GT1 patients with baclabuvir in a phase 2 trial [78]. In another phase 3 trial of an IFN-free regimen containing beclabuvir, a PI and an NS5A inhibitor, around 8% of patients experienced virologic failure which was associated with P495 substitutions in NS5B, in addition to other NS3 and NS5A mutations [79]. Other indole derivatives in development include MBX-700 and MBX-701 (Microbiotix), which are in phase 1, and preclinical development, respectively (Figure 2).

**T2 inhibitors.** Compounds that have been identified as Thumb II (T2) binders include thiophene-2-carboxylic acids [80] like lomibuvir (VX-222) and GS-9669. Both lomibuvir and GS-9669 have progressed to phase 2 clinical trials for the treatment of GT1 infections, however lomibuvir was recently halted from further development. Thiophene-2-carboxylic acids have shown potent antiviral activity *in vitro* [81] and *in vivo* [82,83]. Another class of molecules with T2 specificity is dihydropyranones which include PF-00868554 (filibuvir) [84]. Filibuvir has been shown to have potent antiviral activity both *in vitro* [85] and *in vivo* against GT1 viruses [86], however the development of this agent was halted for commercial reasons. Resistance to T2 inhibitors, lomibuvir and filibuvir, has been demonstrated *in vitro* and most commonly associated with RdRp amino acid substitutions at L419, M423, M426 and I482 (Table 1) [85,87,88]. These substitutions have been shown to cause a loss in the binding affinity of these molecules to the RdRp [87]. Interestingly, M423 substitutions did not have a significant impact on the potency of GS-9669 *in vitro*, however, similar to lomibuvir and filibuvir, resistance was associated with L419M, I482L and R422K [81]. In the clinical setting, amino acid substitutions at positions L419, R422, M423, I482, A486, and V494 (Table 1) were all selected for after monotherapy of patients with GT1 infection using lomibuvir [89]. These substitutions conferred resistance to lomibuvir when examined using the replicon model, but were also associated with reduced replication capacity [89]. In a phase 2 trial of lomibuvir combined with telaprevir (a protease inhibitor), significant viral breakthrough (close to 25%) was observed without Peg-IFN/RBV, and was associated with known lomibuvir resistance variants, including L419S and R422K [89,90]. The study concluded that such regimens (PI/NNI) have an overall low barrier to resistance, and therefore additional agents are required for effective treatment of GT1 infections [90]. Filibuvir administration as monotherapy resulted in 2.3 log10 reduction in HCV RNA in patients infected with GT1, however mutations that encode for M423 substitutions were detected in the majority of patients (76%) after receiving filibuvir at the effective dosage [91]. Of the T2 inhibitors, GS-9669 has shown particularly promising results in two IFN-free regimens for the treatment of both treatment-naïve and experienced patients, with no detectable mutations in a patient that had virologic relapse [83].

**P1 inhibitors.** Inhibitors that bind to the Palm I (P1) site of the RdRp include some of the most advanced HCV NNIs to date. Benzothiadiazine [92] derivatives, such as, dasabuvir (ABT-333), RG7790 (setrobuvir) and ABT-072 have been identified as RdRp P1 binders. Dasabuvir is the first HCV NNI to be approved for the treatment of patients with GT1 infection. In a phase 3 clinical trial of a combination regimen containing dasabuvir, >95% SVR rates were observed after 12 weeks of treatment [93]. The lack of response to treatment as well as viral relapse, which occurred in a minority of patients at 0.2% and 1.5%, respectively, were associated with the P1 substitution S556G [93]. Similarly, both viral breakthrough and relapse in an IFN-free trial including dasabuvir were associated with substitutions at RdRp residues C316, M414, G554, S556 and D559 (Figure 3, Table 1) [94]. Setrobuvir (also known as RG7790 and ANA598) was recently halted from clinical development after reaching phase 2 clinical trials. In combination with Peg-IFN/RBV, setrobuvir has shown potent antiviral activity for GT1 patients [95]. In IFN-free regimens, an SVR rate of 96% has been reported for GT1b patients, but was lower for GT1a (70% [96]). Resistance to setrobuvir has been associated with variants at residues M414, G554 S556 and D559 (Figure 3) [97,98]. The RdRp P1 site has also been targeted by proline [99] and benzodiazepine [100] derivatives.

**P2 and P-β inhibitors.** Benzofurans, including nesbuvir, remain the only class of molecules that have been reported to bind the Palm II (P2) site of the HCV RdRp [41,63]. Despite the potential of these inhibitors, very few reports have further described the development of compounds from the same scaffold, and almost all studies examining the P2 RdRp site have focused on the compound Nesbuvir [101]. Nesbuvir demonstrated promising results *in vitro* [102] and has progressed to phase 2 clinical trials. However, the administration of nesbuvir to HCV infected patients resulted in elevation of liver enzymes, and the compound was halted from further clinical trials [69]. In cell culture models, mutations encoding substitutions at C316, S365 and M414 all conferred resistance to nesbuvir, and this was attributed to reduced affinity of mutant RdRps to the compound [102].

The P-β site of HCV RdRp is the latest site to be identified for NNI binding, after a class of compounds, imidazopyridines, were discovered as palm-binding inhibitors of the RdRp [67]. Resistance analysis of the most advanced compound of this class, GS-9190 (tegobuvir), identified mutations within the palm domain as well as in the β hairpin (Figure 3) which conferred resistance to tegobuvir in HCV replicon models [68]. Later it was found that a metabolite of tegobuvir binds HCV RdRp at the palm domain but interacts with the β-hairpin extending from the thumb domain [67,68]. Resistance to tegobuvir in cell culture models was attributed to RdRp substitutions C316Y, Y448H, Y452H, and C445F (Figure 3) [68]. In the clinical setting, tegobuvir progressed to phase 2 clinical trials. However, the administration of tegobuvir with Peg-IFN/RBV did not have a significant impact on SVR rates when compared to Peg-IFN/RBV alone [103]. In a trial of tegobuvir with a protease inhibitor, GS-9256, the majority of GT1a patients developed double-resistance mutations for these DAAs, with Y448H detected in seven out of eight GT1a patients, while RAVs C316Y and C445F were also detected in two GT1b patients [104]. The clinical development of tegobuvir was recently halted; however, an *in vitro* study recently demonstrated the potential of tegobuvir as a DAA due to its additive effect when examined with PIs or NNIs in inhibiting replicon models and significantly hindering the development of resistance mutations [105].

## 5. Mechanism of Action for RdRp NNIs

The mechanism of inhibition for the HCV NNIs appears to vary depending on which of the allosteric sites the inhibitor binds to, although structural studies indicate that most NNIs interfere with conformational changes required for an initiation and elongation competent RdRp [65]. For instance, benzimidazole and indole derivatives (T1), which have been shown to inhibit the initiation of RNA transcription, had no effect once the RdRp-RNA complex was formed, indicating that these molecules act at a step prior to the formation of a productive RdRp-RNA complex, which is time-dependent [77,106,107]. Crystallographic analyses revealed that these molecules prevent the formation of intramolecular contacts between thumb and fingers domain, preventing the formation of a productive RdRp-RNA complex [108]. The mechanism by which T2 binders inhibit the activity has also been examined extensively. T2 inhibitors have been shown to reduce the affinity of the RdRp to template RNA [109], and these molecules are thought to bind the closed conformation of the enzyme [110,111]. Interestingly, a two-step interaction model has been reported for lomibuvir whereas a single-reversible model has been reported for filibuvir, giving lomibuvir a longer, more efficient interaction and inhibition of the HCV RdRp when compared to other T2 inhibitors [109]. We recently reported a novel activity for molecules that bind both T2 and P-β sites of the RdRp. Surprisingly, we found these NNI actually enhanced the *de novo* activity of the RdRp at increased concentrations, but inhibited primer-dependent transcription in a dose-dependent manner when examined using recombinant enzymes [112]. The enhancement of *de novo* enzyme activity was unexpected given that these molecules were developed as inhibitors of the HCV RdRp. We therefore proposed a model where such molecules stabilise RdRp conformations that are critical for *de novo* activity, overcoming rate-limiting steps in the initiation of RNA transcription. In the context of P-β binder tegobuvir, this was also surprising given that tegobuvir is known to undergo an intracellular activation step by the host cell before it interacts with and inhibits NS5B [113], and is thought to have no effect on the recombinant RdRp without such activation [109].

Palm binders have been suggested to stabilise the β-hairpin extending into the RdRp active site [114,115]. In accordance with these notions, the binding of the RdRp to the RNA had a minor effect on affinity of benzofurans (P2) towards the RdRp [116], and both benzofurans and benzothiadiazines (P1) have been shown to inhibit the two modes of RdRp activity (*de novo* and primer-dependent) [117]. Furthermore, benzothiadiazines have been shown to cause the inhibition of phosphodiester bond formation in both primed and *de novo* [118]. These findings emphasize the importance of different RdRp conformations on the biochemical activity of the enzyme, and the differential effects HCV NNIs have on RdRp conformational changes.

## 6. Genotype Coverage of RdRp Inhibitors

Historically, the development of the first HCV RdRp inhibitors was focused towards GT1 infections for a number of reasons: Firstly, GT1 remains the predominant genotype in most developed countries (~75%) [119]. Secondly, for decades infections with other genotypes responded better to IFN-based therapies (~80% SVR rate for GT2 and GT3, compared to ~50% for GT1 patients) [34]. Thirdly, until recently, models for *in vitro* study of HCV were largely limited to the GT1 and GT2 replicon models, and the GT2 infectious model [120]. For the most part, the relative lack of non-GT1 targeted RdRp DAA development has significantly impacted usefulness of these molecules to treat such infections. Antiviral development has therefore focused on the “harder to treat” and more prevalent GT1 infections, with the hope that the newly discovered molecules will have cross-genotypic inhibitory activity. Despite these factors, as HCV NIs target the active site of the RdRp which is highly conserved between genotypes, they have pan-genotypic activity, at least *in vitro* [44,53]. Surprisingly, however a significant reduction in response to the most advanced NI, sofosbuvir, has been observed in patients with HCV GT3a infection. For reasons not fully understood, when given to patients sofosbuvir was significantly less effective against GT3 compared to all other genotypes [37]. GT3 patients were even less responsive than patients with GT2 infection (62% SVR rate for GT3 *vs.* 94% for GT2 [39], and 56% for GT3 *vs.* 97% for GT2 [37]). However, the addition of sofosbuvir to the IFN-based SOC improves response rates for GT3 patients [40]. Extending treatment duration of sofosbuvir in IFN-free regimens to 24 weeks also significantly improves SVR rates for patients with GT3 infection (up to 85%) [36].

A number of reports have detailed the efficacy of all five known classes of HCV NNIs across HCV GTs *in vitro* [44,112,121,122]. Of all the NNIs that bound the five described allosteric pockets, only benzofurans, which bind to RdRp site P2 (nesbuvir), are cross-genotypic—as demonstrated using recombinant RdRp and replicon models [44,53,112,121,122,123]. T1-binding Indole derivatives like TMC647055 and BMS791325 share a similar inhibitory profile with a notable reduction in potency against GT2 viruses [75]. There is limited data on the efficacy of deleobuvir across HCV genotypes; however, GT1a patients are less responsive to deleobuvir when compared to patients infected with GT1b [74], which is also observed in replicon models of HCV [73]. Benzothiadiazine compounds (P1), also have limited genotype coverage, with limited efficacy against non-GT1 viruses [122,124], and reduced efficacy against GT1b when compared to GT1a *in vitro* [53] The recently licensed dasabuvir, for example, is >400-fold less potent against all non-GT1 HCV RdRps [125]. Similarly, compounds that bind T2 have limited pan-genotype potency, and are generally restricted to GT1 [81,112]. However, thiophene derivatives retain some activity when examined using GT5a HCV models [44,81,123]. The reduced potency of T2 inhibitors in GT2, GT3 and GT4 is thought to be due to conformational changes in this binding pocket due to substitutions I482L and L419I [81]. Overall, all NNIs of the HCV RdRps in current development have limited cross-genotypic activity. This, combined with their lower barrier to resistance, at least compared to NI, poses significant implications for the use of NNIs in future all-oral, pan-genotypic therapies. Dasabuvir (P1 binder) has indeed shown promise as part of a combination treatment for GT1 patients. Whether other NNIs in the near future will be specifically used for certain genotypes remains unclear.

**Table 1:** Prevalence of known HCV RdRp variants associated with resistance to NI and NNIs in treatment naïve patients.

RdRp Site	Resistance Substitutions	Prevalence of Resistant Variants (%)		Refs.
		GT1a	GT1b	
**NI**	S96T	0	0	[126,127,128,129,130]
	L159F	ND	ND	
	S282T	0–0.6	0–1.1	
	L320I/F	0	0	
	V321I	0.19–3.1	2.51–3.3	
**NNI-T1**	T389S/A	ND	ND	
	L392I	ND	ND	
	A421V	0–17.84	0–6.28	
	P495A/L/S	0	0	
	P496A/S/T	0	0–0.84	
	V499A ^#^	94.4–100	5–14.23	
**NNI-T2**	L419M/V/I	0	0–0.9	
	R422K	0–0.56	0	
	M423T/I/V	0–2.8	0–0.42	
	M426T/V	0–3.1	0–6.6	
	I482L/V/T	0–0.2	0–0.3	
	A486V	0	0	
	V494I/A	0	0–0.8	
**NNI-P1**	C316Y/N *	0–0.19	10.88–36.6	
	M414T/L *	0–0.38	0–1.68	
	G554D	0	0	
	S556G	0–0.4	0–8.2	
	D559G/N	0–0.57	0	
**NNI-P2**	S365T/N	0	0	
**NNI-P-β**	C445F	0	0–0.42	
	Y448C/H	0–3.1	0–1.26	
	Y452H	0–3.1	0.42–3.3	

* Also associated with resistance to P2 and P-β binders; ^#^ V499A results in a minor reduction of T1 NNI potency; ND, not determined.

## 7. Prevalence of RAVs in Treatment Naïve Patients

The natural prevalence of RAVs in untreated HCV infections has been analysed by a number of studies, both by population sequencing [126,127,128,129,130], and using next-generation sequencing (NGS) [131,132,133,134]. The majority of these studies, however, focused on infections with GT1 HCV, and the prevalence of RAVs in non-GT1 infections remains poorly characterized. Overall, amino acid variants associated with resistance to NIs in clinical development are rarely observed in untreated HCV patients, even at a very low frequency in the quasispecies population, as determined by NGS [132]. High fitness costs conferred by these resistance mutations are thought to prevent their emergence in untreated populations [57,131]. In addition, the mutational bias of the viral polymerase against transversion events is believed to contribute towards the relative low abundance of NI-associated RAVs, such as S96T and S282T, that require transversion mutations [135].

In contrast to NIs, RAVs associated with NNIs are generally more prevalent due to the relatively low fitness cost associated with these variants and the subsequent higher natural genetic diversity observed in the areas targeted by NNIs (Table 1) [46]. In fact, some of the RAVs against NNIs are naturally present as the dominant variant in GT2a and GT3a infections. These include A499, I419, V424, L482, G556 and F445. HCV NNIs developed against GT1 that have reduced potency against these RAVs have therefore been shown to be less potent against these viruses [112]. For patients infected with GT1, a natural resistance variant at RdRp position 499 has also been observed. The variant A499, which over 96% of GT1a isolates carry, results in a minor shift of the potency of T1 NNIs [130,133,136]. The A499 RAV is also observed as the dominant variant in up to 14% of patients infected with GT1b [126,127,128,130]. Similarly, RdRp variants at position C316, which are associated with resistance to P1, P2 and P-β inhibitors, have been shown to be dominant in 11%–37% of GT1b infections, but are rarely observed in GT1a [126,127,128,130]. Variations in consensus RdRp sequences that are associated with resistance to most other NNIs could also be detected, although at lower frequencies, and depending on the geographical location (Table 1). Furthermore, the prevalence for these RAVs appears to be variable between different GT1 subtypes. For instance, M423 (T2) substitutions are more commonly detected in GT1a infections compared to GT1b, whereas S556 (P1) substitutions is more prevalent in GT1b infections (Table 1). Studies utilising NGS technologies have further analysed the prevalence of HCV RAVs within and between GT1 infections. Results from these studies are concordant with population-based sequencing data for high prevalent variations; however analysis of low abundance variants (present at levels below 20% of the quasispecies) revealed that these RAVs are present, albeit at low frequency, in a large proportion of infections. On average, 30%–40% of infections carried low-abundance resistance variants [131,132,133,134], however, while up to 75% of GT1 patients carried low-abundance variants at positions such as M423, M494 and M423, similar variants were not detected for other positions such as M482 and P496 [131,132], which could be explained by the reduced fitness cost of such variants [127]. The impact of low-abundance RAVs on the outcome of treatment with HCV NNI remains poorly understood, but equivalent variants appeared to have minimal effect on protease inhibitor therapies [137]. As more NNIs move into clinical settings and NGS analysis of the viral quasispecies continues to become more accessible, the effect of low-prevalence variation at resistance sites will become clearer.

## 8. Conclusions

Understanding the structure and function of the HCV RdRp has led to the development of the most successful antiviral for chronic infections to date. In addition to sofosbuvir, several other NIs and NNIs are in clinical development, and the recently approved NNI dasabuvir is the first of its class to arrive on the market. These compounds differ in their potency, mechanism of action, and genotype coverage. Combined with other promising NS3 and NS5A inhibitors, efficacy will continue to improve across genotypes. At the population level, treatment cost remains a major barrier. Additionally, multiple personal, social, and health infrastructure barriers remain for injecting-drug users to successfully access and complete antiviral therapy. Hence, it remains to be determined whether significant treatment scale-up is affordable, achievable and effective. Finally, as these inhibitors become more accessible, the impact of antiviral resistance will likely become clearer, particularly in regimens containing NNIs.
